# Survey on health literacy and related factors among firefighters of emergency management departments in Southwest China

**DOI:** 10.1186/s12889-024-19522-6

**Published:** 2024-07-24

**Authors:** Peijie Jiang, Quanxin Chen, Ruifeng Liu, Tingchun Peng, Haili Zhao, Jian Chen, Junguo Xin, Xiaohong Yang

**Affiliations:** 1 School of Public Health, Chengdu Medical College, Chengdu, Sichuan Province China; 2 Department of Epidemiology and Biostatistics, School of Public Health, Chengdu Medical College, Chengdu, China

**Keywords:** Health literacy, Firefighters, Emergency management department, Related factors, Health promotion, Southwest China

## Abstract

**Objective:**

Although health literacy (HL) has emerged as a critical public health concern, research on HL in emergency management departments is limited. This study aimed to investigate the awareness of HL and associated factors among firefighters of emergency management departments in southwest China to provide a basis for carrying out targeted health education.

**Methods:**

A cross-sectional convenience sample of 1,742 firefighters from an emergency management department in southwestern China was surveyed from February to April 2023 using the Chinese Citizen’s Health Literacy Questionnaire (2019 version). The chi-square test, linear trend chi-square test, Fisher’s test, rank sum test, and multifactorial logistic regression model were used to identify influential factors associated with HL.

**Results:**

The HL level of the 1742 respondents was 34.3%. Age, ethnicity, education level, length of service, type of job, smoking status, types of parental jobs, annual household income, time of daily internet use, etc. (*P* < 0.05). The results of multivariate logistic regression analysis indicate that type of job (OR = 0.648, 95%CI:0.426–0.985), length of service (OR = 0.496, 95%CI:0.251–0.981), household income (OR = 1.900, 95%CI:1.443–2.502), daily internet usage time (OR = 0.726, 95%Cl:0.588–0.896), health status (OR = 0.750, 95%Cl:0.585–0.962) and frequency of organizing HL sessions (OR = 1.603, 95%Cl:1.101–2.330) were influencing factors affecting the HL of the officers and soldiers.

**Conclusion:**

The health literacy level of firefighters in the Emergency Management Department in Southwest China was 34.3%. Lower levels were found in the health-related skills dimension (HRS, 30.1%) and in infectious disease control (ID, 30.7%). Health information literacy (HI, 34.3%) was lower than the national level. The type of urban and rural areas, literacy level, and household income level may be the factors affecting the level of health literacy among the respondents. Therefore, health education and promotion interventions should target high priority dimensions (HRS, HI, and ID) and should focus on strengthening health literacy levels of firefighters with rural types, low education levels, and low household income to improve their health.

## Introduction

Health literacy is the degree to which individuals can find, understand, and use information and services to inform health-related decisions and actions for themselves and others. Health literacy involves more than reading — it also includes specific skills, such as calculating the right dose of a medicine, following directions for fasting before a surgery, or checking a nutrition label to make sure an item is safe for someone with a food allergy. People with low health literacy skills may have trouble doing these things [1]. Several studies in recent years have shown that health literacy has attracted increasing attention from researchers and policymakers, suggesting that health literacy may play an important role in maintaining or improving health and is considered an important factor in health promotion and disease prevention [2]. Health is an inevitable requirement for the promotion of all-around human development and a basic condition for economic and social development. The Chinese government is paying increasing attention to the health literacy level of its citizens, which has reached 29.70% in 2023 based on a survey [3]. To understand the level of health literacy of the population, countries have conducted health literacy tests. For example, the 2022 study showed high levels of health literacy among the Portuguese [4]. A survey of health literacy among the general population in eight European countries showed that 53% of the population was health literate, with significant differences in health literacy levels between countries [5]. In Chile, 83.40% of the population was found to be health literate [6]. In a study of the German resident population aged 18 and over, 41.20 per cent were found to be health literate [7]. Therefore, effectively improving HL has become a significant challenge for many countries and an important public health goal worldwide.

The Ministry of Emergency Management of the People’s Republic of China is a comprehensive permanent emergency rescue team formed by the joint merger of the former Public Security Fire Force, the Armed Police Forestry Force, and the Safety Production Emergency Rescue Team [8]. Emergency management firefighters have been maintaining high-intensity training and strict military management mode for a long time, and are responsible for epidemic, earthquake, fire, floods and other major safety and first aid tasks, even in the ordinary work of life is also at risk. However, Southwest China is located in China’s earthquake-prone areas, as well as fire-prone areas, Southwest China’s emergency rescue management firefighters face even more onerous tasks. Therefore, understanding the health literacy level of firefighters and its related influencing factors is essential to promote the health and well-being of this group of people as well as to improve their emergency response capabilities [9, 10]. However, research on HL in this population is limited. Understanding the awareness of HL and its associated factors among emergency firefighters is critical for promoting their health and well-being and improving their emergency response performance. To address this gap, we conducted a cross-sectional survey to investigate the awareness of HL and its associated factors among firefighters in emergency management departments in Southwest China. This study aimed to provide insights into the status of HL among emergency management departments and to identify potential factors that may influence HL in this population. The findings can support the development of targeted interventions to improve HL and health outcomes among firefighters in the emergency management department.

## Methods

### Study population and sampling design

In this study, 1,742 emergency rescue firefighters were selected from the firefighters of emergency rescue departments in Southwest China from February to April 2023, based on a cross-sectional research design and convenience sampling method. The survey included active fire fighters and commanders, with no age or gender restrictions. New trainees less than three months old were excluded, as well as those who were unwilling to participate in this survey. Referring to the literature method [11], the estimated health literacy level was taken as 30%, and the permissible error δ for the subjects of this study was taken as 2.50%, *α* = 0.05, and *µ*_*е/2*_ = 1.96. According to the formula (1–1), the required sample size was 1290. the effective sample size of this study was 1742 firefighters.

1-1 $$a = {\mu _{/2}}^2 \times p \times 1 - p/{\delta ^2}$$

### Questionnaire and measure of health literacy

Two instruments used in the study were the “Demographic Information Survey”, which focuses on demographic characteristics, including sex, age, ethnicity, marital status, education level, type of work, age at work, smoking and alcohol consumption, and the In this study, we referred to the health literacy scoring standards for Chinese residents published by the China Health Education Centre [2], and used the Chinese Citizen Health Literacy Questionnaire (HLQ) (2019 edition), which developed by the Chinese Ministry of Health, had strong internal consistency (Cronbach’s a = 0.95) and folded-half reliability (Spearman- Brown correlation coefficient of 0.94) [11]. These questions identified three dimensions of health literacy: knowledge and attitudes (KAA); knowledge and attitudes toward health-related behavior and lifestyle (BAL); health-related skills (HRS); scientific views of health (SVH); infectious diseases (ID); chronic diseases (CD); safety and first aid (SAFA); medical care (MC); and health information (HI). The questionnaire was scored out of 66 points and consisted of 50 questions including judgement, single choice, multiple choice, and situational questions. Judgement questions and single choice questions will be awarded 1 mark for correct answers and no marks for mistakes; multiple choice questions will be awarded 2 marks for correct answers to all options.2 points for correct answer of all options, no points for multiple choice, omission, wrong choice. A score of 80% or more of the total score (53 and above) was considered to be health literate. and the ratio of the number of health literate people to the total number of people in the study was considered to be the overall health literacy level of the research population. Similarly, for different dimensions or aspects of literacy, a score of 80 per cent or more of the total score was considered as having health literacy in that dimension, and the ratio of the number of people having each dimension to the total number of corresponding study participants represented the level of health literacy in that dimension [2, 11, 12].

### Quality control

The design stage of the survey programme was conducted through the review and analysis of health literacy-related literature and the actual situation of the selected survey site, which in turn determined the purpose, content, methodology and specific implementation of the survey study. Strict pre-training was given to the investigators to unify the survey standards and methods, and the investigators explained the purpose of the survey to the respondents face-to-face in detail, so as to allay their concerns as much as possible, and to improve the degree of co-operation and the quality of questionnaire responses. After obtaining the informed consent of the respondents, the respondents were asked to answer the questionnaires in the form of centralised assessment on formal occasions and at formal times, so as to avoid any error bias due to personal negative attitudes or subjective desires. A uniform questionnaire was used, in an anonymous format, to ensure the authenticity and reliability of the questionnaire. Two data analysts collated, classified and screened the questionnaires and analysed the results.

### Statistical analyses

Data were analyzed using Stata MP version 17.0. Descriptive statistics were used to analyze the distribution of HL in three dimensions and six aspects and expressed as the mean ± SD. The chi-square test, linear trend chi-square test, Fisher test, and rank-sum test were used to compare HL levels among sociodemographic characteristics, health-related behaviors, and family-related factors. After examining the correlation between variables and controlling for covariates, a binary logistic regression was used to determine the relationship between HL levels, sociodemographic characteristics, and health factors. At the same time. Statistical significance was considered for two-tailed *P* < 0.05.

## Results

### Demographic characteristics

A total of 1822 people were surveyed, and 1742 valid responses were obtained; the response rate of the questionnaire was 95.6%. Among the survey respondents, there were more firefighters than fire commanders (92.1% vs. 7.9%). In terms of education level, 1227 (70.4%) held a college degree, bachelor’s degree or above. A large proportion of the participants (78.4%) lived in rural areas, and participants with a household income of 30,000 to 80,000 yuan ($4,254 to $11,320) per year were the largest income group (38.2%); 630 (36.2%) attended health knowledge classes monthly in their workplace (Table 1).

**Table 1:** Association of HL levels with sociodemographic characteristics, health-related behaviors, and family-related factors of 1742 respondents

Characteristic	*N*	*N* of > 80% score	HL levels (%)	χ^2^|Z	*P*
**Sex**					
Male	1629	563	34.6	0.603	0.474
Female	113	35	31.0		
**Age group (years)**					
18–21	210	67	31.9	12.966	0.005
22–25	876	331	37.8		
26–29	410	114	27.8		
≥ 30	246	86	35.0		
**Nation**					
Han	1003	347	34.6	13.394	0.009
Miao	182	67	36.8		
Zhuang	116	24	20.7		
Dong	121	51	42.2		
Other Minorities	320	109	34.1		
**Education**					
Junior High School and below	26	9	34.6	27.900^b^	<0.001
High School and Secondary School	489	116	23.7		
College, undergraduate and above	1227	473	38.5		
**Type of job**					
Fire Commander	183	75	41.0	4.017	0.048
Fire Fighter	1559	523	33.5		
**Length of service (years)**					
≤ 2	894	357	39.9	18.658^b^	<0.001
2–5	359	103	28.7		
5–10	387	106	27.4		
≥ 10	102	32	31.4		
**Place of residence**					
Rural	1365	441	32.3	11.423	0.001
Urban	377	157	41.6		
**Household income (10**,**000 yuan** **[$1**,**415] per year)**					
< 30,000	539	185	24.5	29.673^b^	<0.001
30,000–79,999	666	255	38.3		
80,000-149,999	395	143	36.2		
150,000-299,999	122	57	46.7		
≥ 300,000	20	11	55.0		
**Type of father’s occupation**					
Farmer	1216	379	31.2	32.752	<0.001
Worker	198	72	31.2		
State civil servant or career worker	124	49	39.5		
Military personnel	3	0	0		
Professional technician	13	4	30.8		
Businessman	94	41	43.6		
Others	53	53	56.4		
**Type of mother’s occupation**					
Farmer	1295	404	31.2	26.687^a^	<0.001
Worker	171	76	44.4		
State civil servant or career worker	76	27	35.5		
Professional technician	5	4	80.0		
Businessman	95	41	43.2		
Others	100	46	46.0		
**Daily internet usage time (hours)**					
≤ 1	593	260	43.8	19.205^b^	<0.001
1–2	536	167	31.2		
2–3	190	48	25.3		
3–4	168	40	23.8		
≥ 4	255	83	32.5		
**Frequency of searching for HI**					
Hardly	291	89	30.6	10.358^b^	0.001
Sometimes	1165	385	33.0		
Often	286	124	43.4		
**Credibility of online HI**					
Very credible	99	29	29.3	9.334^b^	<0.001
Fairly credible	489	206	42.1		
Fair	1046	336	32.1		
Relatively untrustworthy	81	23	28.4		
Very untrustworthy	27	4	14.8		
**Smoking status**					
Daily	483	143	29.6	8.398^b^	0.018
Occasionally	182	55	30.2		
Used to smoke	263	100	38.0		
Never smoked	814	300	36.9		
**Drinking status**					
Drink daily	19	4	21.1	0.194^b^	0.405
Occasionally	736	256	34.8		
Used to drink	459	166	36.2		
Never drink	528	172	32.6		
**Health status**					
Good	691	284	41.1	50.278^b^	<0.001
Better	652	233	35.7		
Fair	349	68	19.5		
Poor	50	13	26.0		
**Frequency of organizing health** **literacy sessions**					
Hardly	252	67	26.6	26.892	<0.001
Once a week	324	102	31.5		
Once a month	630	264	41.9		
Once a quarter	536	165	30.8		

^a^ Fisher’s test

^b^ Linear trend χ² test

### Awareness of the three dimensions and six aspects of health literacy

The three dimensions of health literacy were 45.1% for basic knowledge and concepts, 43.5% for healthy lifestyles and behaviours, and 30.1% for health skills. The six dimensions of literacy were 66.6% for scientific health concepts, 30.7% for infectious disease prevention and control, 46.8% for chronic disease prevention and management, 79.9% for safety and first aid, 34.2% for basic medical care and 34.3% for health information literacy (Table 2).

**Table 2:** Average score and level of the three dimensions and six aspects of HL.

Topics	Respondents’ achievements			Respondents’ level (%)	National level (%)	χ^2^	*P*
	Mean ± SD	Valid	Invalid				
**Three Dimensions**							
KAA	20.01 ± 4.77	785	957	45.1	37.7	39.109	<0.001
BAL	15.96 ± 4.38	758	984	43.5	28.1	197.946	<0.001
HRS	10.60 ± 3.40	524	1218	30.1	24.3	30.715	<0.001
**Six Aspects**							
SVH	8.05 ± 2.34	1160	582	66.6	50	186.912	<0.001
ID	4.63 ± 1.56	535	1207	30.7	27.6	8.209	0.004
CD	8.83 ± 2.56	816	926	46.8	26.7	347.771	<0.001
SAFA	11.77 ± 2.92	1392	350	79.9	56.4	382.782	<0.001
MC	9.03 ± 2.89	595	1147	34.2	26.1	56.846	<0.001
HI	4.26 ± 1.81	361	1381	34.3	35.9	170.751	<0.001
**HL**	46.57 ± 11.46	598	1144	34.3	25.4	70.995	<0.001

**Abbreviations**: BAL, basic knowledge and attitudes toward health-related behavior and lifestyle; CD, chronic diseases; HI, health information; HL, health literacy; HRS, health-related skills; ID, infectious diseases; KAA, knowledge and attitudes; MC, medical care; SAFA, safety and first aid; SVH, scientific views of health

### The relationship between health literacy levels and the characteristics of participants

The overall health literacy HL level of 1,742 emergency unit personnel was 34.3%. People with higher education or above showed higher HL levels than those with lower education levels. The HL level of urban firefighters is 41.6%, which is significantly higher than the 32.3% of rural firefighters(*P* < 0.05). Firefighters’ HL level was 33.5% lower than the 41.0% of fire commanders(*P* < 0.05). Firefighters with high annual household incomes had significantly higher levels of HL than groups with low yearly household incomes(*P* < 0.05). Those in excellent health had better HL levels than those in poor health(*P* < 0.05). In addition, we found higher HL levels for officers and enlisted personnel who had regular health education courses. The results of this study showed that several factors were significantly associated with HL (*P* < 0.05), such as age, ethnicity, education level, length of service, type of work, type of parental occupation, urban and rural household registration, annual household income, time spent using the Internet per day, frequency of searching for health knowledge and credibility of online information, smoking status, health status, and frequency of organizing health education classes. However, there were no significant differences in HL levels between sex, marital status, and alcohol consumption. (Table 1).

### Multiple logistic regression analysis of factors influencing health literacy

According to the multiple logistic regression analysis results, firefighters were found to have a 0.648 higher likelihood of having adequate HL than fire commanders (95% CI, 0.426–0.985). Similarly, those with a length of service of more than ten years were 0.496 times more likely to have adequate HL than those with less than two years of work service (95% CI, 0.251–0.981). In terms of annual household incomes, using people with an annual household income below RMB 30,000 as a reference, the likelihood of having adequate HL increased significantly with increasing annual household income, with ORs of 1.9, 2.083, 3.874, and 4.749, respectively. Time spent on the Internet per day was also one of the significant influencing factors of HL level, with ORs of 1–2 h, 2 h, 3 h, and more than 4 h being 0.559 (95% CI, 0.415–0.754), 0.417 (95% CI, 0.267–0.651), 0.363 (95% CI, 0.227–0.582), and 0.569 (95% CI, 0.379-0. 855), respectively. In addition, we also found that those who occasionally searched for and frequently searched for health information were 1.163 and 1.863 times more likely to have adequate HL than those who hardly searched for health information, respectively. Monthly health education in the unit was 1.603 times more likely to result in adequate HL than almost no health education (95% CI, 1.101–2.330) (Table 3).

**Table 3:** Multiple logistic regression analysis of factors influencing health literacy in 1742 respondents

Characteristic	β	S.E.	Wald	*P*	OR	95% CI
**Age group (years)**						
18–21	Reference					
22–25	0.139	0.193	0.520	0.471	1.150	(0.787 ~ 1.680)
26–29	0.066	0.253	0.068	0.794	1.068	(0.650 ~ 1.756)
≥ 30	0.539	0.309	3.049	0.081	1.714	(0.936 ~ 3.138)
**Nation**						
Han	Reference					
Miao	0.153	0.191	0.636	0.425	1.165	(0.800 ~ 1.695)
Zhuang	-0.305	0.268	1.301	0.254	0.737	(0.436 ~ 1.245)
Dong	0.340	0.224	2.302	0.129	1.404	(0.906 ~ 2.177)
Other Minorities	-0.080	0.152	0.279	0.597	0.923	(0.685 ~ 1.243)
**Education**						
Junior High School and below	Reference					
High School and Secondary School	-0.570	0.467	1.492	0.222	0.565	(0.226 ~ 1.412)
College, undergraduate and above	0.151	0.460	0.108	0.743	1.163	(0.472 ~ 2.863)
**Type of job**						
Fire Fighter	Reference					
Fire Commander	-0.434	0.214	4.115	0.043	0.648	(0.426 ~ 0.985)
**Length of service (years)**						
≤ 2						
2–5	-0.082	0.186	0.195	0.658	0.921	(0.640 ~ 1.326)
5–10	-0.249	0.200	1.554	0.213	0.779	(0.527 ~ 1.153)
≥ 10	-0.701	0.348	4.064	0.044	0.496	(0.251 ~ 0.981)
**Place of residence**						
Rural	Reference					
Urban	-0.002	0.177	< 0.001	0.989	0.998	(0.705 ~ 1.412)
**Household income (10**,**000 yuan** **[$1**,**415] per year)**						
< 30,000	Reference					
30,000–79,999	0.642	0.140	20.883	< 0.001	1.900	(1.443 ~ 2.502)
80,000-149,999	0.734	0.172	18.126	< 0.001	2.083	(1.486 ~ 2.921)
150,000-299,999	1.354	0.261	26.830	< 0.001	3.874	(2.321 ~ 6.467)
≥ 300,000	1.558	0.506	9.476	0.002	4.749	(1.761 ~ 12.805)
**Type of father’s occupation**						
Farmer	Reference					
Worker	-0.246	0.245	1.009	0.315	0.782	(0.484 ~ 1.264)
State civil servant or career worker	0.088	0.299	0.085	0.770	1.091	(0.607 ~ 1.963)
Military personnel	-21.522	22524.466	< 0.001	0.999	< 0.001	(0.000)
Professional technician	-1.131	0.862	1.723	0.189	0.323	(0.060 ~ 1.747)
Businessman	0.256	0.354	0.522	0.470	1.291	(0.646 ~ 2.582)
Other	1.420	0.330	18.540	< 0.001	4.136	(2.167 ~ 7.894)
**Type of mother’s occupation**						
Farmer	Reference					
Worker	0.690	0.265	6.808	0.009	1.995	(1.187 ~ 3.350)
State civil servant or career worker	-0.247	0.365	0.459	0.498	0.781	(0.382 ~ 1.597)
Professional technician	1.773	1.439	1.519	0.218	5.888	(0.351 ~ 98.745)
Businessman	-0.099	0.354	0.078	0.780	0.906	(0.453 ~ 1.812)
Others	-0.239	0.329	0.529	0.467	0.787	(0.413 ~ 1.500)
**Daily internet usage time (hours)**						
≤ 1	Reference					
1–2	-0.581	0.153	14.509	< 0.001	0.559	(0.415 ~ 0.754)
2	-0.876	0.228	14.797	< 0.001	0.417	(0.267 ~ 0.651)
3	-1.013	0.241	17.690	< 0.001	0.363	(0.227 ~ 0.582)
≥ 4	-0.563	0.208	7.364	0.007	0.569	(0.379 ~ 0.855)
**Frequency of searching for HI**						
Hardly	Reference					
Sometimes	0.151	0.162	0.876	0.349	1.163	(0.847 ~ 1.597)
Often	0.622	0.205	9.181	0.002	1.863	(1.246 ~ 2.787)
**Credibility of online HI**						
Very credible	Reference					
Fairly credible	0.539	0.261	4.263	0.039	1.715	(1.028 ~ 2.861)
Fair	0.352	0.254	1.921	0.166	1.422	(0.864 ~ 2.341)
Relatively untrustworthy	0.432	0.368	1.376	0.241	1.541	(0.748 ~ 3.171)
Very untrustworthy	-0.549	0.633	0.751	0.386	0.578	(0.167 ~ 1.998)
**Smoking status**						
Daily	Reference					
Occasionally	-0.148	0.213	0.481	0.488	0.863	(0.568 ~ 1.309)
Used to smoke	0.194	0.184	1.115	0.291	1.214	(0.847 ~ 1.740)
Never smoked	0.215	0.138	2.415	0.120	1.240	(0.945 ~ 1.625)
**Health status**						
Good	Reference					
Better	-0.288	0.127	5.135	0.023	0.750	(0.585 ~ 0.962)
Fair	-0.956	0.178	28.853	< 0.001	0.384	(0.271 ~ 0.545)
Poor	-0.922	0.380	5.871	0.015	0.398	(0.189 ~ 0.839)
**Frequency of organizing health literacy sessions**						
Hardly	Reference					
Once a week	-0.006	0.222	0.001	0.980	0.994	(0.644 ~ 1.535)
Once a month	0.472	0.191	6.114	0.013	1.603	(1.101 ~ 2.330)
Once a quarter	0.079	0.190	0.172	0.678	1.082	(0.746 ~ 1.570)
**Constant**	-1.117	0.643	3.015	0.083	0.327	

**Abbreviations**: B, beta; CI, confidence interval; HI, health information; HL, health literacy; OR, odds ratio; SE, standard error

## Discussion

### Emergency Management Department Health Literacy Levels

According to the results of China 2023 monitoring, the health literacy level of Chinese residents reached 29.70%. This paper reports on a survey of the health literacy levels of 1,742 first responders in Southwest China. The survey found that the health literacy level of this personnel was 34.3%, which was higher than the national average and exceeded the 30% level required in the Health China 2030 Plan Outline [13]. Looking at the urban and rural resident types, both urban and rural personnel were well above the national level.

### Health literacy in Emergency Management: three dimensions and six types of concern

Looking at the three dimensions and six categories of questions, the current respondents compared to the national level in 2021 [14], KAA (45.1% vs. 37.66%), BAL (43.5% vs. 28.05%), HRS (30.1% vs. 24. The firefighters were above the national level in all three dimensions: SAFA (79.9% vs. 56.41%), SVH (66.6% vs. 50.01%), HI (34.3 vs. 35.93%), ID for prevention and control (30.7 vs. 27.60%), CD for prevention and control (46.8% vs. 26.67%) and MC (34.2 vs. 26.05%). only for health information were emergency responders below the national level in all six categories. This may reflect officers and servicemen living in closed training environments with limited access to health information [15].

The results found that the emergency management department firefighters’ health literacy was uneven, the ability to perform basic KAA and BAL was high, and the mastery of HRS was low, indicating that most firefighters have high health-related knowledge and healthy lifestyles but relatively insufficient mastery of health-related skills. The reason is that under the management system of the military, workers have a fairly good routine with long and intense daily training, a regular lifestyle, good habits for health-related behaviors and lifestyles and excellent knowledge and attitudes toward health [16]. However, heavy training usually leads to a lack of learning of the theory of health-related skills; perhaps the lack of attention given to it by firefighters leads to a low level of mastery of health-related skills [15].

### Factors influencing health literacy

Univariate analyses showed differences in appropriate health literacy between urban and rural residents, but after adjusting for other demographic factors, this association was no longer detected. The possible reason for this is that urban residents have a higher level of education than those in rural areas, and the correlation between education level and health literacy is stronger. Similar to related reviewed research, most research found differences in health literacy between urban and rural areas, but after considering the influence of other factors, half of the studies found that rural areas themselves were not a significant influence on health literacy levels, and there were no differences between urban and rural areas after controlling for covariates. Rural areas were not the only cause of health literacy differences and were influenced by other factors [17].

The health literacy level of ethnic minorities in the study was low, and ethnic minorities other than the Han Chinese accounted for a total of 42.4% of the subjects in this study, suggesting that the firefighters’ workforce is rich in ethnic cultures, which may be due to the fact that the Southwest region of China is a place where ethnic minorities gather [18]. The health of ethnic minorities is also one of the most popular concerns of researchers [19]. China has the largest number of ethnic minorities in the world, and different ethnic groups may differ in basic skills due to the influence of their ethnic cultures, as well as their living environments, and the health literacy level of ethnic minorities is also affected by other factors. Relevant studies have concluded that low economic income, poor health, limited access to medical services, and different traditional concepts about the causes of disease and biomedicine contribute to the high proportion of ethnic minorities with low health literacy [17, 19–23].

In addition, in terms of annual household incomes, using people with an annual household income below RMB 30,000 as a reference, the likelihood of having adequate HL increased significantly with increasing annual household income because those from wealthy families usually have a rich knowledge base. They have a strong ability and opportunity to access and understand health information and to use it in their daily lives. People with high levels of education also have a higher ability to learn than those with low levels of education [24]. Education level was the most important factor influencing health literacy, with a positive linear correlation between health literacy and education level. Those with a university degree or higher have a higher level of health literacy than those with lower levels of education. In addition, household income was identified as influencing health literacy levels. Higher income leads to better health awareness, better housing and living conditions, reduced life stress, and improved diet quality [25]. Higher levels of health literacy have been reported to be associated with higher household income [26, 27], similar to our results. Health literacy awareness Health literacy rates were lower among participants with lower household incomes. Lower levels of household income. As household income increases, participants may pay more attention to self-health management and quality of life.

As with previous research, time spent using the Internet was correlated with health literacy levels, and appropriate use of the Internet to learn health-related knowledge and skills is one way to improve health literacy levels; the use of the Internet as a strategy for accessing medical information is very encouraging [28].

In terms of job type, commanders had higher health literacy than combatants (*P* < 0.05) because commanders all graduated from higher command colleges and received higher and more comprehensive education, a result that once again reflects the importance of the level of education, which is not high and comprehensive, resulting in lower levels of health literacy. Health education should consider personnel’s different levels of literacy and adopt different strategies for other groups. As in previous research, educational attainment is an important influencing factor in health literacy [29].

The study also found that a lower level of health knowledge was significantly associated with a lower smoking frequency, suggesting that personal health behaviors can directly influence health knowledge levels. According to related review research, behavioral interventions, including smoking prevention, were found to be effective in influencing health literacy levels in 68% of studies, with significant improvements in health literacy after the intervention [30]; simple models of interventions, including instructional animations, 10-minute training videos, and text messages, are equally effective in improving health literacy [31–34]; health literacy interventions can not only influence line change but also learn from behavior change, and interventions designed according to behavior change principles will probably be more effective [30].

Occasional and frequent searchers of health information in this research had ORs 1.163 and 1.863 times more likely to be health literate than those who hardly searched for health information. Using Internet tools around us to retrieve health information was a contributing factor to improving health literacy levels, and similarly, in a survey of Shanghai residents, it was found that the use of traditional media and the Internet and participation in offline activities were more helpful for residents to obtain health information and improve their health literacy level [35]. Regarding how to increase residents’ attention to health information, a study from China found that posts with new, informative headlines focusing on chronic diseases, nutrition and food-borne illnesses, and infectious diseases were more popular [36]. Analyses of the results of our research showed that in the unit, monthly health education was held 1.603 times (95% CI, 1.101–2.330) more than in healthy people with almost no health education who had adequate HL. Improving the overall quality of the emergency management department requires first strengthening education.

### How can the health literacy of firefighters be improved?

Health education should focus on personal health behaviors, and interventions should be implemented to improve the health awareness and skills of personnel, such as recruiting more college students, appropriately increasing the time spent on the Internet to learn health knowledge, increasing the frequency of conducting health education knowledge lectures and strengthening the focus on personal health management in training. Education should not be limited to the interior of the emergency management unit but can be outwardly joined with local hospitals, CDCs, and medical schools for long-term cooperation and regular health talks for firefighters to expand access to health knowledge as well as training health-related skills. To improve population health literacy, K. Sørensen et al. propose a framework that includes the construction of eight action areas, including a health literate workforce, a health literate organization, health literate data governance, person-centered services, a user engagement-based environment, health literate leadership, health literacy investments and financial resources, health literate-based technology and innovation, partnerships and intersectoral collaboration, to reduce the adverse effects of inadequate population health literacy levels [37]. Y. Gao et al. proposed a multichannel, multimodal, and multisectoral collaboration to build a health education model that meets actual needs to improve public health literacy, with government departments as the main focus and medical institutions and the media as the supplement [38]. The German study found that an integrated healthcare system might be helpful in the area to improve the health literacy of the region’s population [39].

### Limitations

Overall, this study has some reference value in understanding the health literacy levels and influencing factors of emergency rescue force officers and soldiers and provides some insights and suggestions for strengthening the health literacy of force officers and soldiers. However, several limitations should be acknowledged. First, the sample of this study was limited to some of the emergency management department workers in the southwest region, and the results may need to be more generalizable and validated in a broader sample. Second, The comparability of this study comparing this respondent’s health literacy level with the national health literacy level is low because data on education level, family income, and age group subgroups of the National Health Literacy Survey have not been found.; Third, although the questionnaire we used to measure health literacy was comprehensive, comparisons of health literacy status with those of other countries are difficult because of different reference standards for health literacy.

## Conclusion

The health literacy level of firefighters in the Emergency Management Department in Southwest China was 34.3%. Of the three dimensions of health literacy, health-related skills (HRS, 30.1%) had the lowest level; of the six dimensions of health literacy, infectious diseases (ID, 30.7%) had the lowest level. Health information (HI, 34.3%) was below the national level. Levels of health literacy in the prevention and control of communicable diseases and primary health care were low and need to be strengthened. The type of urban and rural areas, level of education, and level of household income may be the factors influencing the health literacy level of the respondents. Therefore, health education and promotion interventions should target high priority dimensions (HRS, HI, and ID) and should focus on strengthening health literacy levels of firefighters with rural types, low levels of education, and low household incomes to improve health. It is recommended that emergency management departments increase health literacy courses or hospitals and community health departments increase health literacy education for emergency responders to further improve soldiers’ health literacy.

## Data Availability

The data that support the findings of this study are available from [Emergency Management Department, Southwest China] but restrictions apply to the availability of these data, which were used under license for the current study, and so are not publicly available. Data are however available from the authors upon reasonable request and with permission of [Emergency Management Department, Southwest China].
